# Ibandronate Reduces the Surface Bone Resorption of Mandibular Bone Grafts: A Randomized Trial With Internal Controls

**DOI:** 10.1002/jbm4.10468

**Published:** 2021-02-10

**Authors:** Jahan Abtahi, Benjamin Klintström, Eva Klintström

**Affiliations:** ^1^ Department of Oral & Maxillofacial Surgery and Department of Biomedical and Clinical Sciences Linköping University Linköping Sweden; ^2^ Center for Medical Image Science and Visualization (CMIV) Linköping University Linköping Sweden; ^3^ Department of Biomedical Engineering and Health Systems KTH Royal Institute of Technology Stockholm Sweden; ^4^ Department of Radiology and Department of Health, Medicine and Caring Sciences Linköping University Linköping Sweden

**Keywords:** BISPHOSPHONATE, BONE GRAFT, BONE HEALING, BONE RESORPTION, OSTEOCLAST

## Abstract

Autologous bone grafts are considered the gold standard for reconstruction of the edentulous alveolar ridges. However, this procedure is associated with unpredictable bone loss caused by physiological bone resorption. Bisphosphonates are antiresorptive drugs that act specifically on osteoclasts, thereby maintaining bone density, volume, and strength. It was hypothesized that the resorption of bone grafts treated with an ibandronate solution would be less advanced than bone grafts treated with saline. Ten patients who underwent bilateral sagittal split osteotomy were included in a randomized double‐blind trial with internal controls. Each patient received a bone graft treated with a solution of ibandronate on one side and a graft treated with saline (controls) contralaterally. Radiographs for the measurement of bone volume were obtained at 2 weeks and at 6 months after surgery. The primary endpoint was the difference in the change of bone volume between the control and the ibandronate bone grafts 6 months after surgery. All of the bone grafts healed without complications. One patient was excluded because of reoperation. In eight of the nine patients, the ibandronate bone grafts showed an increase in bone volume compared with baseline, with an average gain of 126 mm^3^ (40% more than baseline) with a range of +27 to +218 mm^3^. Only one ibandronate‐treated graft had a decrease in bone volume (8%). In the controls, an average bone volume loss of −146 mm^3^ (58% of baseline) with a range of −29 to −301 mm^3^ was seen. In the maxillofacial field, the reconstructions of atrophic alveolar ridges, especially in the esthetical zones, are challenging. These results show that bone grafts locally treated with ibandronate solution increases the remaining bone volume. This might lead to new possibilities for the maxillofacial surgeons in the preservation of bone graft volumes and for dental implant installations. © 2021 The Authors. *JBMR Plus* published by Wiley Periodicals LLC. on behalf of American Society for Bone and Mineral Research.

## Introduction

The irreversible physiological resorption of alveolar bone occurs as early as 3–6 months following tooth extraction, tooth loss, or dental aplasia.^(^ ^1^ ^)^ In the maxillofacial regions, the reconstruction of atrophic jawbone might be challenging because of an unpredictable and relatively high resorption rate. Various surgical techniques have been described for the reconstruction of bone defects, including onlay block grafting, particulate bone grafting, and guided bone regeneration.^(^ ^2^, ^3^ ^)^ Autogenous bone graft is considered the gold standard in treating an atrophic alveolar ridge caused by its osteogenesis, osteoconduction, and osteoinduction abilities compared with other bone substitutes.^(^ ^4^ ^)^ However, an increase in intervention morbidity, unpredictable bone resorption at the recipient site, and limited intraoral bone volume are limitations of this bone‐grafting procedure.^(^ ^5^ ^)^

Currently, a variety of regional or distant donor sites are being used, including iliac crest bone, intraoral bone, proximal tibial bone, costal bone, and calvarium.^(^ ^6^, ^7^, ^8^, ^9^, ^10^, ^11^ ^)^ Mandibular bone harvesting is associated with less resorption compared with a bone graft of the iliac crest, which has been attributed to the iliac graft's relatively higher trabecular structure.^(^ ^6^, ^12^ ^)^ The reported resorption rates for cortical onlay grafts of the iliac crest and particulate inlay grafts of trabecular bone for the reconstruction of maxillary bone defects were approximately 50%.^(^ ^6^ ^)^ The resorption rates of a symphyseal mandibular graft augmenting in the anterior maxilla are estimated to be 25% after 4 months and 60% after 10 months.^(^ ^12^ ^)^ The use of inorganic bovine bone and barrier membranes might decrease graft resorption after implantation and maintain graft volume over time.^(^ ^13^ ^)^

Bisphosphonates are pharmaceuticals in clinical use that have been tested extensively for treating osteoporosis.^(^ ^14^ ^)^ Bisphosphonates are also part of the current pharmacological arsenal against bone loss caused by Paget disease of bone, malignancies metastatic to bone, hypercalcemia, and multiple myeloma.^(^ ^14^ ^)^ These drugs act specifically on osteoclasts for maintaining bone volume, density, and strength.^(^ ^15^ ^)^ Locally applied bisphosphonate reduces peri‐implant resorption allowing orthopedic and dental implants to achieve a stronger primary fixation.^(^ ^16^, ^17^, ^18^ ^)^ This has been shown in both clinical and experimental studies.^(^ ^19^, ^20^, ^21^ ^)^ In a series of clinical and radiological studies, fibrinogen‐coated dental implants with the immobilized bisphosphonates, pamidronate and ibandronate, were inserted into human maxillas. Coated implants had better implant stability and less marginal bone resorption.^(^ ^21^, ^22^, ^23^ ^)^ Moreover, the local treatment of periodontitis with a gel containing a very high concentration of bisphosphonate (alendronate) was found to be successful in regenerating a large part of the bone loss, whereas the placebo had little effect.^(^ ^24^ ^)^

Reconstruction of atrophic alveolar ridges is challenging for implant practitioners because of a high resorption rate, especially in the esthetical zones. This might be reduced by the use of locally administered bisphosphonates. Therefore, we hypothesized that mandibular bone grafts treated with an ibandronate solution would show less resorption than the controls.

## Materials and Methods

Patient data regarding age at the time of surgery, sex, and surgical complications were collected from medical records. Oral and written information was provided, and written consent was obtained. The study was approved by the Regional Ethical Board in Linköping, Sweden (2015/85‐31), and was monitored by the Linköping Academic Research Center, which reviewed all of the procedures during the study and checked the data before data lock and unblinding. This study was also approved by the Swedish Medical Products Agency (EudraCT‐number 2014‐003817‐28).

### Data source and study design

This study was a prospective randomized controlled double‐blind trial. Over a 4‐year period (October 2015 to March 2020), 10 patients aged 19 to 57 years (mean age, 24 years; six men and four women) were included. The patients were scheduled for bilateral sagittal split osteotomy; all of the patients underwent orthodontic treatment by a consultant in orthodontics. Facial skeletal deformities of the maxilla and mandible included overgrowth (hyperplasia) and undergrowth (hypoplasia), as well as asymmetries. The inclusion criteria were (i) retrognathia/overbite, which refers to an abnormal posterior positioning of the maxilla or mandible, particularly the mandible; (ii) prognathism/underbite, which refers to an extension or bulging out (protrusion) of the lower jaw; (iii) an open bite; and (iv) mandibular laterognathia/mandibular asymmetry. All patients had a complete series of identifiable lateral cephalograms and panoramic radiographs, and sufficient maxillary bone (classes I‐II according to Cawood and Howell^(^ ^25^ ^)^. Healthy patients without a history of tooth extraction participated in this randomized study. Therefore, the degree of mandibular atrophy was considered to be minimal.

The exclusion criteria were systemic or immunologic disease, drug abuse, uncontrolled diabetes, smoking, previous tumor, trauma, or surgery in the mandible region. We also excluded patients with ongoing or previous treatments known to effect bone metabolism, such as steroid drugs, antiresorptive drugs, immunosuppressive drugs, or hormonal therapy.

**Fig 1:**
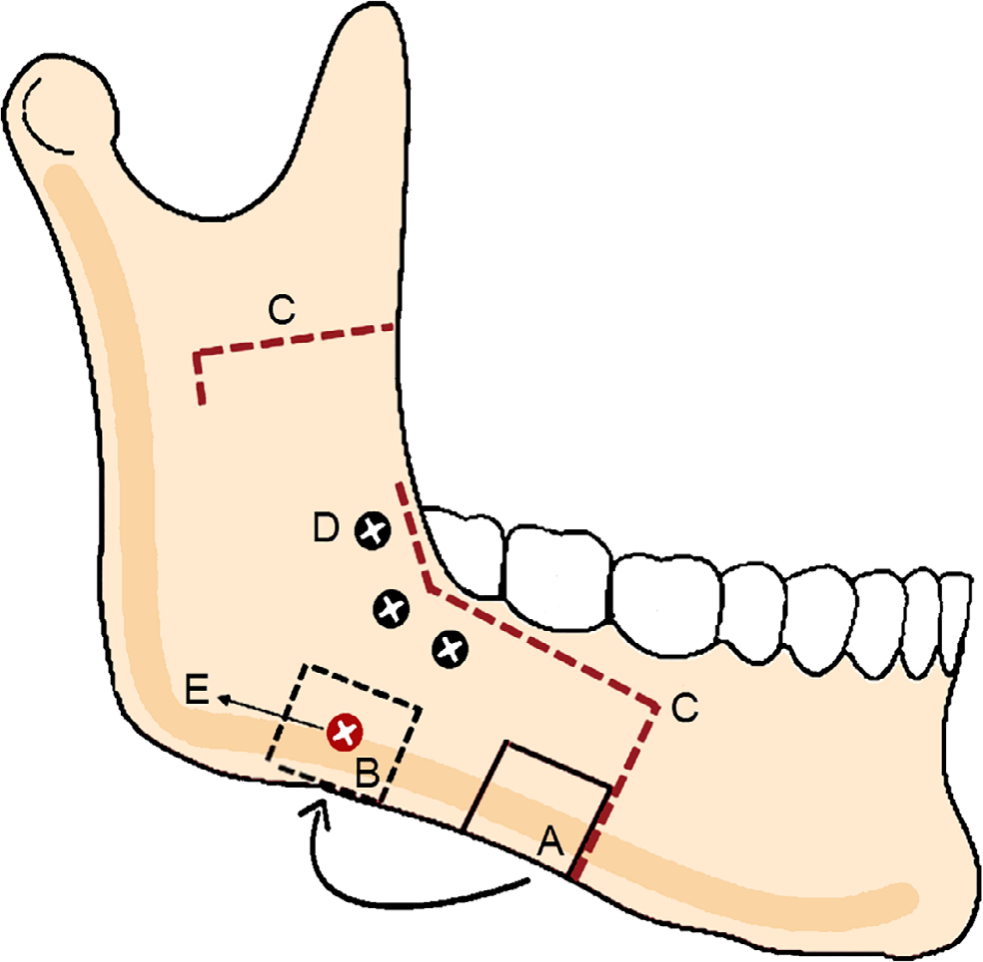
Schematic photo of surgical and anatomical structures. (*A*) Bone graft donor site at the inferior border of lateral segment of mandible split. (*B*) Bone graft recipient site at the mandible angle. (*C*) Osteotomies line of mandibular sagittal split (dashed red line). (*D*) Position screw for the fixation of medial and lateral segment. (*E*) Position screw for fixation of bone graft.

In all patients, osteotomies were performed according to Dal Pont and colleagues.^(^ ^26^ ^)^ Bone grafts were taken from the anterior part of the lateral segments of the mandible with a fissure bur, and each patient received a bone graft treated with ibandronate solution on one side and saline on the contralateral side, following a standardized protocol. The bone grafts were then fixed on the buccal surface of the mandible angle with a titanium position screw (Fig. 1). Preoperatively, all patients used mouth rinse consisting of a chlorhexidine solution (hexident 0.20%; Meda AB) to minimize the risk of a postoperative infection. The effect of this procedure has been shown in a previous study.^(^ ^27^ ^)^ All other treatment and follow‐up procedures were carried out according to clinical routines. Cone beam computed tomography (CBCT) imaging was used for baseline and control examinations. Orthognathic surgery is associated with considerable swelling and limited mouth opening. Therefore, the first radiological examination (baseline) was conducted 2 weeks after the surgery. The follow‐up radiological examination was scheduled for 6 months (26 weeks) after the surgery.

### Surgery

The surgery was performed by the first author (JA) at the Department of Maxillofacial Surgery at Linköping University Hospital. Bilateral inferior alveolar nerve blocks with local anesthetic and vasoconstrictor were given (lidocaine dental adrenalin injection solution 20 mg/ml, 12.5 μg/ml). A 3g prophylactic penicillin G was administered by IV 15 minutes before the surgical incision; this was repeated every 8 hours on the day of the operation. Patients with a history of penicillin allergy were given 600 mg clindamycin. The mucosa, submucosa, muscle, and periosteum were incised with electrocautery continuing posteriorly, from the external oblique ridge to the first molar.

Dissection was carried out by a periosteal elevator (Obwergeser). The inferior alveolar nerve at the entrance of the mandibular canal was located and protected by a retractor, and Kocher forceps were placed to provide superior retraction. The osteotomies were performed according to the technique of Trauner and Obwegeser in 1957^(^ ^28^ ^)^ and modified by Dal Pont in 1961.^(^ ^26^ ^)^ Briefly, a lingual horizontal osteotomy and a vertical osteotomy on the buccal side between the second premolar and first molar was carried out using a Lindeman fissure bur and a thin‐fissured high‐speed bur. A reciprocating saw was then placed to the ascending ramus, superior to the lingula and parallel to the occlusal plane, and the cut continued anteriorly from the posterior site of the external oblique ridge to the level of the first molar. The split maneuver was performed by using original Stille‐design chisel with a 6‐mm and 8‐mm tip. The occlusion was stabilized via wafer and temporary intermaxillary fixation was initiated with 0.4‐mm wires. A bone block (~10 × 10 mm) was then grafted from the anterior site of the proximal segment at each site of the mandible. The bone grafts were treated with either ibandronate solution or saline and fixed with a titanium screw to the mandible, approximately 5 mm from the mandible angle and approximately 5 mm from the inferior border of the mandible (Fig. 1). The proximal segment was positioned into the correct position in the temporomandibular fossa. The two segments were then fixated to each other by three bicortical screws. A miniplate with three holes on either side of the osteotomy was used if there was instability between the proximal and medial segments. The incisions were sutured with nonabsorbable sutures following copious irrigation and hemostasis. Guiding elastics were placed intraoperatively.

### Randomization procedure

The bone grafts were taken from the right and left side of the mandible. A nurse outside the room, who was otherwise not involved in the treatment or study, opened a nontransparent randomization envelope. She then treated either the right bone or left bone graft with ibandronate, depending on the instructions in the envelope. For all bone grafts (10 sites) in the ibandronate group, 1 ml of a 6 mg/ml solution of an ibandronate (Bondronat; Roche AB) was used. The other bone grafts (10 sites) were treated with a Ringer's acetate solution for infusion 9 mg/ml (Braun Melsungen AG). The ibandronate and saline solutions were visually indistinguishable. Six Gallipot, sterile 150‐ml clear plastic cups (three for the right and three for the left bone graft) were placed on the surgical instrument table and numbered 1 to 3. For the ibandronate group, the first plastic cup contained a solution of 100‐ml Ringer's acetate and 1‐ml (6 mg) ibandronate. The second and third plastic cups only contained 100‐ml Ringer's acetate. The nurse put the bone grafts in the first plastic cup for 3 minutes and in the second and third cups for 15 seconds each. For the saline group, all the plastic cups contained 100 ml Ringer's acetate. The bone grafts were then fixed with a titanium position screw to the mandible. After delivering the bone grafts, the nurse placed a paper with the patient's personal identity number and name in the envelope and sealed it. The envelopes were then stored by the monitor until data lock and unblinding. Thus, the trial was performed in a double‐blinded fashion, with the exception of the otherwise uninvolved nurse.

### Radiographic examination and measurements

**Fig 2:**
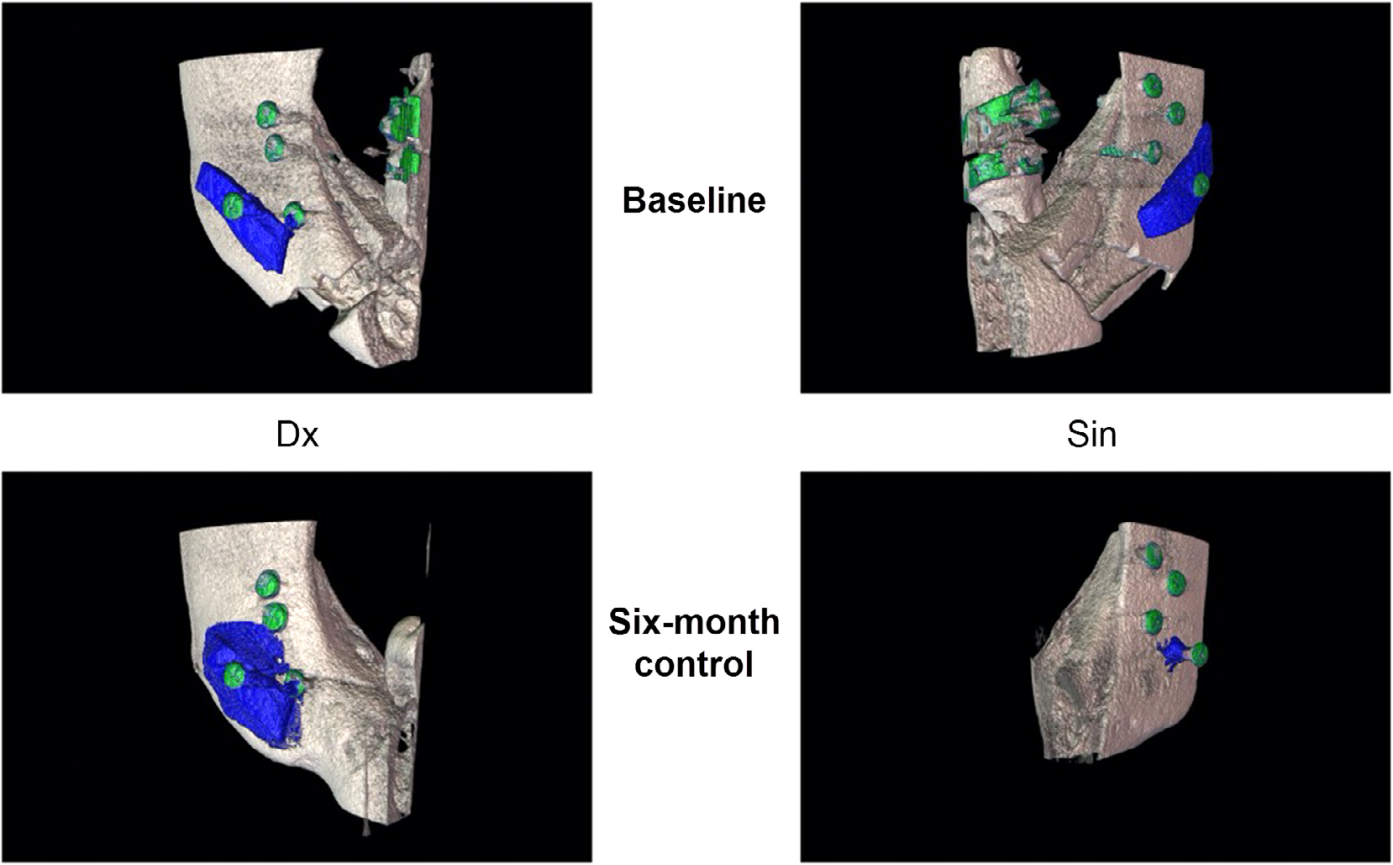
Three‐dimensional images for the patient with ID 6922, showing the results from the segmentation at the baseline and 6‐month control. Segmented bone grafts volumes (blue) and position screws or braces on teeth (green). Right images show the left side of the mandible (Sin) and left images show the right side of the mandible (Dx).

The CBCT scanner used in this study was a 3D Accuitomo 80 (J. Morita MFG). The scanning parameters were: tube voltage = 85 kV, tube current = 5 mA, field of view = 40 mm, voxel size = 80 μm, and exposure time =17 seconds. The second and last authors (BK and EK) performed all of the radiological measurements and analyses. Before segmentation methods were applied, the 6‐month control volumes were registered to the baseline volumes using the Registration Manual‐module in MeVisLab version 3.3 VS2017‐64 (MeVis Medical Solutions AG). After that, a mask was created based on the baseline image to ensure that the mandible and the metal screws were excluded from the resulting segmented volume. Because CBCT‐data do not provide reliable Hounsfield units, the data were normalized to have a mean attenuation of 0 and a SD of 1000. To decrease the processing time, the image data were down‐sampled to a voxel size of 160 μm before being segmented using the Threshold Level Set module in Mia Lab with a Curvature Weighting of 0.6 and low/high threshold of 300/600, respectively.^(^ ^29^ ^)^ The resulting segmentations were then upscaled to the original voxel size of 80 μm. The bone volume of interest was defined as the largest continuous volume of segmented bone within the predefined mask. Results of one of the segmentations at baseline and at the 6‐month control are shown in Fig. 2.

### Statistical analysis

The evaluation was based on internal controls. A descriptive statistical analysis was conducted, and the results were expressed as percentages and/or mean values. Differences in bone volume changes were analyzed by the *t* test for paired data using MatLab version R2020a Update 3 (9.8.0.1396136) 64‐bit version (MathWorks).

## Results

Over a 4‐year period (October 2015 to March 2020), 10 patients 19 to 57 years of age (mean age, 24 years: six men and four women) were included. In this double‐blind, bone‐graft study with internal controls, we found significantly greater bone volumes for the ibandronate‐treated graft sites compared with the contralateral placebo sites. These results were registered at the 6‐month control X‐ray examinations.

Demographic data, diagnoses, and treatment outcomes are shown in Table 1. In the study, 10 patients with a mean age of 24.8 years (range, 18‐57 years), subjected to a bilateral sagittal split osteotomy were included. One patient (ID 8606) was excluded because of reoperation caused by unstable fixation on the placebo side. Besides the incomplete fixation for this patient, there were no surgical complications, no sign of wound infection, and the treatment was clinically judged as successful in all patients.

**Table 1:** Patient Characteristics, Diagnoses, Surgical Treatments, Time Set Up for Surgery, and Radiological Examination

Patient no. & (ID)	Age	Sex	Diagnosis	Treatment	Complications	Surgery date	Radiology baseline	Radiology 6 mo
1 (8096)	18	M	MR	BSSO	No	2015‐10‐27	2015‐11‐10	2016‐04‐26
2 (6754)	57	F	MR	BSSO & genioplasty	No	2016‐04‐06	2016‐04‐19	2016‐09‐27
3 (8837)	21	M	MR	BSSO	No	2017‐06‐09	2017‐06‐26	2017‐12‐05
4 (8606)	24	M	MR	Le Fort I & BSSO	Yes	2018‐03‐14	2018‐03‐29	2018‐10‐02
5 (9304)	19	M	MR	Le Fort I & BSSO	No	2018‐10‐02	2018‐10‐16	2019‐05‐07
6 (1674)	20	F	MR	Le Fort I & BSSO	No	2019‐04‐17	2019‐05‐02	2019‐10‐28
7 (4252)	20	M	MP	Le Fort I & BSSO	No	2019‐05‐29	2019‐06‐12	2019‐12‐10
8 (8323)	19	F	MR	Le Fort I & BSSO	No	2019‐08‐28	2019‐09‐11	2020‐02‐24
9 (5421)	22	M	MP	Le Fort I & BSSO	No	2019‐10‐09	2019‐10‐22	2020‐05‐07
10 (6922)	20	F	ML	BSSO	No	2020‐03‐04	2020‐03‐20	2020‐09‐22

Abbreviations: BSSO = bilateral sagittal split osteotomy; ML = mandibular laterognathia; MP = mandibular prognathism; MR = mandibular retrognathia.

All of the results are based on the remaining nine patients (18 bone grafts). The mean follow‐up time after the 6‐month control was 11.6 months (range, 2‐14 months).

**Fig 3:**
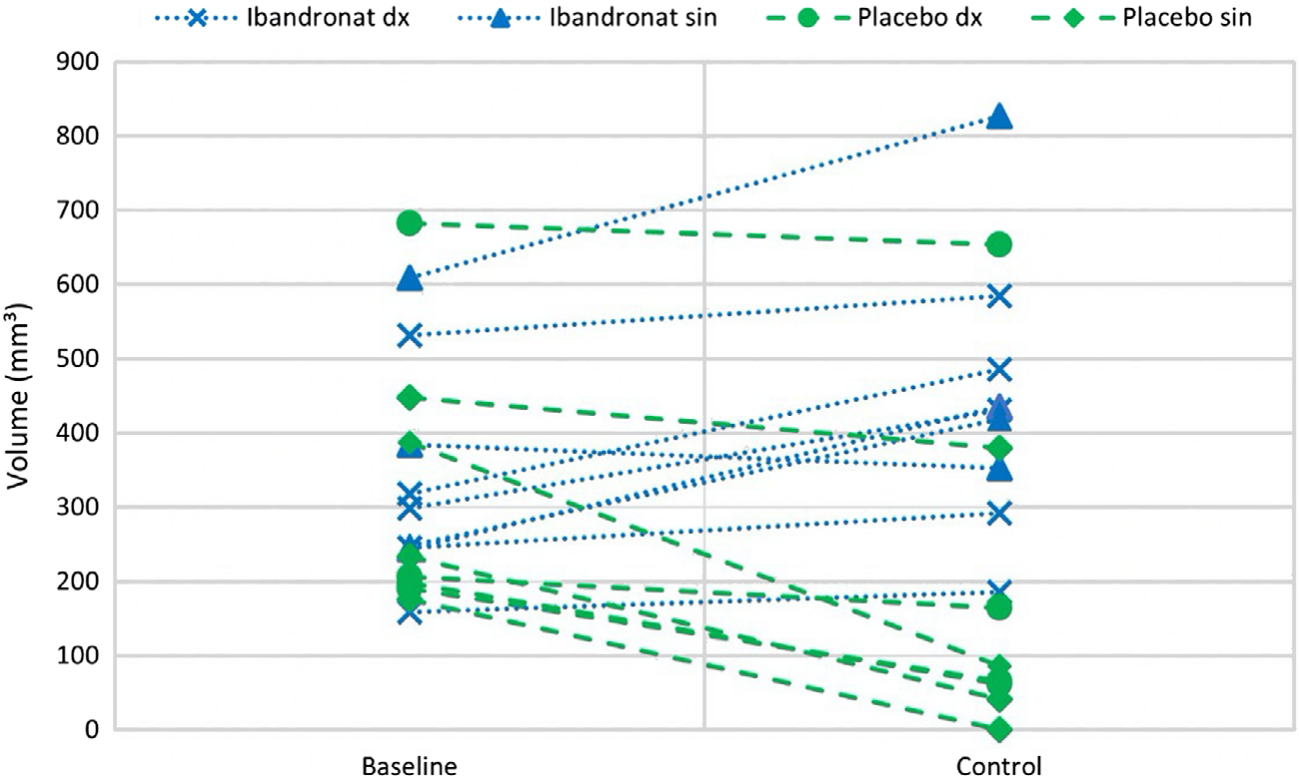
Changes in bone volumes for the ibandronate group and controls. All bone grafts in each patient are connected by a line from baseline to 6‐month control (; blue lines = ibandronate, Dx = right side of the mandible; green lines = controls; Sin = left side of the mandible).

**Fig 4:**
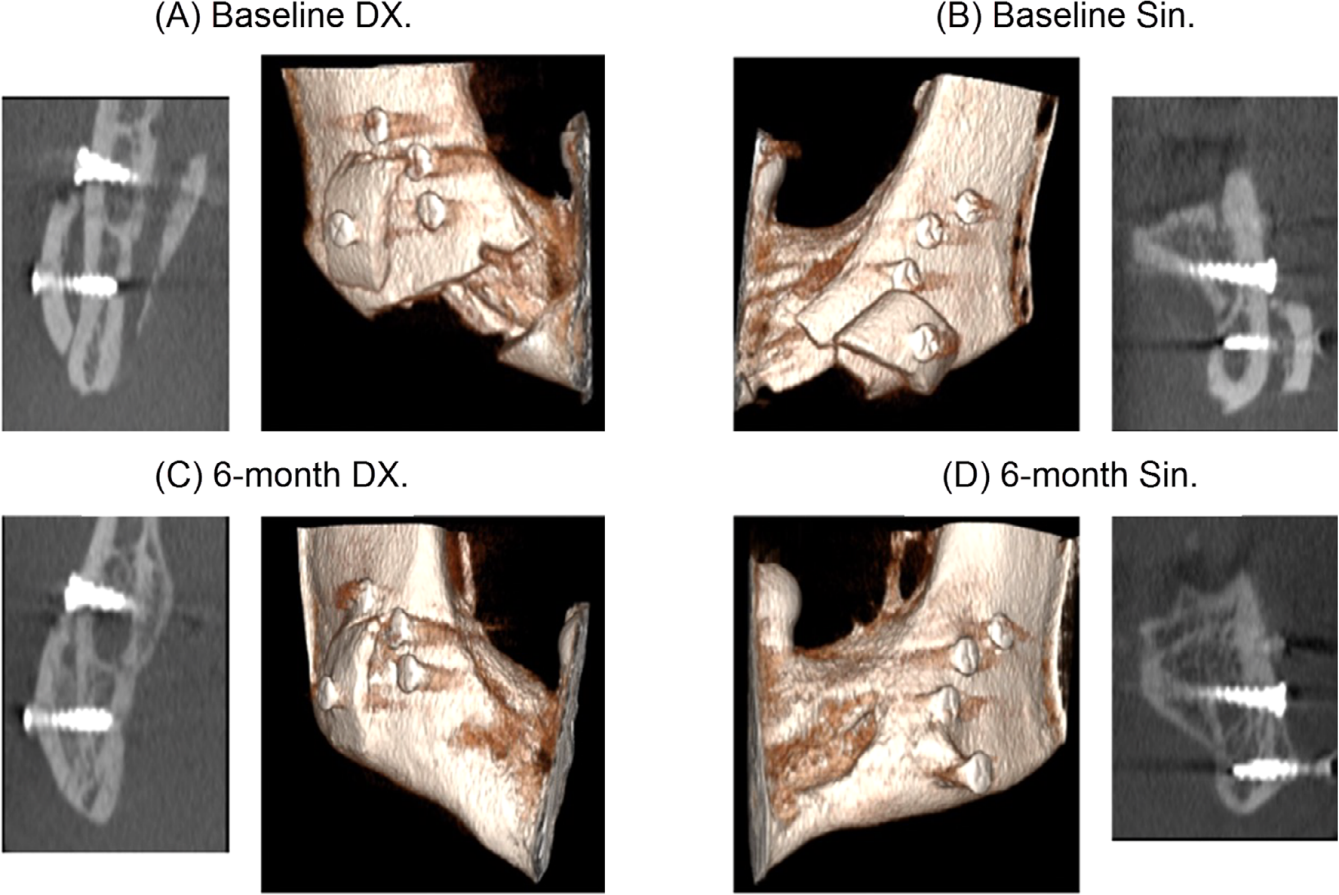
Three‐dimensional volumes and coronal cone beam computed tomography X‐ray slices of patient ID 5421. At the top images from the baseline and below from the 6‐month control. Right images show the left side of the mandible (Sin) and left images show the right side of the mandible (Dx).

Eight of the nine bone transplants treated with ibandronate showed clear increases in volumes (10%–79%) at the 6‐month control X‐ray examinations compared with baseline (Table 2, Fig. 3). Only one of these grafts had a small decrease in volume (8%) compared with baseline. The increase in bone volume could be seen at all sides of the transplants, adapting the transplants to the buccal side of the mandible with a smooth contour. The buccal compact surfaces of the mandibles became integrated with the transplants and changed structurally to become more trabeculated (Fig. 4). All nine contralateral placebo transplants decreased in volume between 4% and 100%. One of the placebo bone grafts was completely resorbed.

**Table 2:** Changes in bone Volumes (mm^3^) for paired bone grafts in 10 patients

Patient no. and (ID)	IB‐site	Baseline volume mm^3^		6 months volume mm^3^		Change in volume mm^3^ and (% of baseline)		Paired diff mm^3^ (IB – C)
		Dx	Sin	Dx	Sin	IB	C	
1 (8096)	Dx	**159**	175	**186**	0	27 (17%)	−175 (−100%)	202 mm^3^
2 (6754)	Sin	206	**243**	165	**436**	192 (79%)	−41 (−20%)	233 mm^3^
3 (8837)	Sin	198	**384**	61	**353**	−31 (−8%)	−136 (−69%)	105 mm^3^
4 (8606)	Dx	**552**	428					
5 (9304)	Dx	**245**	233	**292**	41	46 (19%)	−192 (−82%)	238 mm^3^
6 (1674)	Sin	682	**609**	653	**826**	218 (36%)	−29 (−4%)	247 mm^3^
7 (4252)	Dx	**532**	448	**585**	380	53 (10%)	−68 (−15%)	121 mm^3^
8 (8323)	Sin	190	**248**	65	**419**	171 (69%)	−125 (−66%)	296 mm^3^
9 (5421)	Dx	**318**	387	**486**	86	167 (53%)	−301 (−78%)	469 mm^3^
10 (6922)	Dx	**298**	264	**430**	21	132 (44%)	−243 (−92%)	375 mm^3^
Abbreviations: C = control; IB = ibandronate; Dx = dexter; Sin = sinister.		Mean ± SD (IB at baseline) 337 ± 139 mm^3^		Mean ± SD (IB at 6 mo) 446 ± 172 mm3 *		Mean ± SD (IB) 108 ± 82 (35% of baseline) **		**Mean** ± **SD** 254 ± 108 mm^3^
		Mean ± SD (C at baseline) 309 ± 159 mm^3^		Mean ± SD (C at 6 mo) 164 ± 204 mm^3^*		Mean ± SD (C) −146 ± 87 (−58% of baseline)**		*p = 0.00001 **p = 0.00017
						Cohen's *d* = 2.34		

A comparison of the bone volume changes between the baseline and 6‐month control for the experiment and the control group resulted in a mean difference of 254 mm^3^ (95% confidence interval, 39–469 mm^3^; *p* = 0.00017; Table 2 and Fig. 3).

## Discussion

Autologous intraoral bone grafts are considered the gold standard for the reconstruction of the edentulous alveolar ridge, allowing patients to become candidates for dental implants. However, this procedure is associated with unpredictable bone loss caused by physiological bone resorption. In our study, we found that an ibandronate solution could prevent the resorption of mandible bone grafts. These results represent clinical proof of the possibility to increase bone graft volumes by local use of the drug. All, except one, of the bone grafts treated with ibandronate showed an increase in bone volume from the baseline to the 6‐month control. This is in contrast to the control group, where all of the grafts decreased in bone volume instead.

The reported resorption rates for cortical onlay grafts of the iliac crest for the reconstruction of maxillary bone defects was approximately 50% after a healing period of 6 months.^(^ ^6^ ^)^ Sbordone and colleagues evaluate the long‐term remodeling of autogenous corticocancellous grafted iliac bone followed by dental implant placement.^(^ ^30^ ^)^ The authors used computerized tomographic scans to compare volumes of grafts adjacent to dental implants over time (up to 6 yr). Volumetric measurements of the remaining bone grafts showed a progressive bone resorption of almost all the grafted bone in the maxilla (100%) and mandible (87%). The resorption rates of the symphyseal mandibular graft augmenting the anterior maxilla are estimated to be 25% after 4 months and 60% after 10 months.^(^ ^12^ ^)^ In the present study, 10 patients were subjected to orthognathic surgery, and each patient received a bone graft treated with either ibandronate solution or saline (controls). Nine of the 10 included patients were subjected to radiological analysis (18 sites). One patient underwent a second surgery within 2 weeks after the first surgery and was excluded from the study. A bone graft treated with ibandronate in another patient (patient 3) decreased in volume (−8%). However, this reduction was smaller compared with the control (−69%). In eight patients, the ibandronate bone grafts showed a larger increase in bone volume from baseline to 6 months than did the controls (an average gain of 126 mm^3^; range, +27 to +218 mm^3^; *p* value = 0.00001).

Generally, a bone loss of approximately 50% will be expected within 6 to 12 months after bone grafting regardless of the donor site. In this new study, we found that eight of the nine bone grafts treated with ibandronate instead had increased in bone volume, which is very encouraging.

Bisphosphonates are antiresorptive drugs that specifically act on osteoclasts, thereby promoting the maintenance of both bone density and strength.^(^ ^15^ ^)^ Bisphosphonates have been given orally or systemically to improve the fixation of orthopedic implants.^(^ ^31^, ^32^ ^)^ In a randomized, double‐blind trial of a hybrid‐type total hip arthroplasty in patients with osteoarthritis, Wilkinson and colleagues^(^ ^32^ ^)^ found that a single dose of 90 mg of pamidronate significantly reduced femoral bone loss. Hilding and colleagues showed that the local application of a bisphosphonate during total joint surgery reduced the migration of metal prostheses when measured by radiostereometry.^(^ ^31^ ^)^ In animal models, several investigators have found that systemic bisphosphonates are effective in reducing alveolar bone loss.^(^ ^33^, ^34^, ^35^ ^)^ Furthermore, in a previous clinical trial, we found that dental implants coated with a fibrinogen layer with pamidronate and ibandronate showed considerably better fixation at 6 months after insertion than the uncoated controls.^(^ ^21^ ^)^ The positive results of these previous clinical studies gave us the idea to use local bisphosphonate treatment to preserve bone transplant volume.

Over the last decade, there have been problems with the condition known as bisphosphonate‐related osteonecrosis of the jaw (ONJ). This condition is defined as an area of exposed bone in the maxillofacial region that does not heal within 8 weeks of identification by a health care provider, in a patient who currently receives or has been exposed to a bisphosphonate and has not had radiation therapy to the craniofacial region. It is remarkable that orthopedic surgeons and osteoporosis researchers consider bisphosphonates to be beneficial and useful in many areas, while dental practitioners reject these drugs. Clinically, the disease presents as exposed alveolar bone that occurs spontaneously or becomes evident following a surgical procedure such as tooth removal or dental implant placement.^(^ ^36^ ^)^ These lesions often become symptomatic when surrounding tissues are inflamed or when there is clinical evidence of infection. The incidence of ONJ is estimated to be 1%–12% in cancer patients receiving high‐dose IV bisphosphonates.^(^ ^34^ ^)^ The frequency of ONJ in bone malignancy cases, which were mainly treated with IV bisphosphonates, was found to be 1 in 100.^(^ ^36^ ^)^ If tooth extractions were carried out, the calculated frequency of ONJ was 1 in 10.^(^ ^36^ ^)^ In osteoporosis patients, bisphosphonate‐associated ONJ is rare, and the incidence may not be greater than the natural background incidence of the condition.

In the present study, a potential risk associated with bisphosphonate used dental would be that resorption could be increased in case of infection, whereby the infected bone could be lost, leading to chronic osteomyelitis, similar to ONJ. However, if any such local adverse effects should appear, the problem would be easily solved by removing the bisphosphonate‐containing bone graft in the immediate vicinity of the jawbone.

There are some limitations to this study. First, there is the limited number of bone grafts included. However, the layout of this double‐blind study with internal controls increases the strength of the results substantially. In addition, it was only possible to analyze the results for 9 of the 10 patients (18 sites because it was impossible to align the control and baseline image volumes caused by the reoperation of the patient). This probably did not affect our study results crucially because the differences in bone resorption rates were so large (Table 2).

The use of cortical bone is associated with less resorption compared with cancellous grafts that have the ability to revascularize sooner than cortical grafts because of their trabecular structure.^(^ ^6^, ^12^, ^37^ ^)^ Successful integration of bone grafts depends on factors such as soft tissue thickness, soft tissue coverage, and adequate revascularization. In general, bone grafts at the recipient sites are covered with thin oral mucosa without proximity to any muscle tissue. In this study, the bone grafts were placed at the mandibular angle, adjacent to masseter muscle receiving an adequate blood supply and with a distance far away from the oral mucosa. This might, to some extent, partly explain the positive results of this study. However, it does not explain the differences found here because both the ibandronate and the placebo grafts were treated the same way. To evaluate the above‐mentioned possible limitations, more research is needed to ascertain if our used method also works in alternate situations.

## Conclusion

One of the most common surgical techniques for the reconstruction of atrophic alveolar ridges is autologous intraoral bone grafting. However, a drawback of this method is the unpredictable resorption of bone. In the present study, it is shown that treating bone grafts with ibandronate solution prevents resorption, even leading to an average net gain in the local bone volume. This could lead to new possibilities for the preservation of bone grafts and for dental implant installations.

## Author Contributions

**Jahan Abtahi:** Conceptualization; data curation; formal analysis; investigation; methodology; project administration; supervision; visualization; writing‐original draft; writing‐review & editing. **Benjamin Klintström:** Data curation; formal analysis; software; writing‐review & editing. **Eva Klintström:** Data curation; formal analysis; methodology; software; writing‐original draft; writing‐review & editing.

## Conflict of Interest

The authors declare that there is no conflict of interests that could be perceived as prejudicing the impartiality of the research reported.

## Ethics Statement

The study was approved by the Regional Committee for Ethics in Linköping, Sweden (2015/85‐31), and by the Swedish Medical Products Agency (EudraCT‐number 2014‐003817‐28).

### Peer Review

The peer review history for this article is available at https://publons.com/publon/10.1002/jbm4.10468.
